# RESTORE: an exploratory trial of a web-based intervention to enhance self-management of cancer-related fatigue: findings from a qualitative process evaluation

**DOI:** 10.1186/s12911-015-0214-y

**Published:** 2015-11-14

**Authors:** Michelle Myall, Carl R. May, Chloe Grimmett, Christine M. May, Lynn Calman, Alison Richardson, Claire L. Foster

**Affiliations:** University of Southampton, Faculty of Health Sciences, Southampton, SO17 1BJ UK; University Hospital Southampton NHS Foundation Trust, Southampton, UK

**Keywords:** Process evaluation, Normalisation Process Theory, Cancer survivors, Self-efficacy, Oncology, Cancer

## Abstract

**Background:**

Cancer-related fatigue is a distressing symptom experienced by many after cancer treatment. An exploratory randomised controlled trial was conducted to test proof of concept of RESTORE: a web-based tool to enhance self-efficacy to manage cancer-related fatigue. This paper reports findings from a qualitative process evaluation to determine feasibility and acceptability of the intervention and trial processes.

**Method:**

Qualitative process evaluation carried out at the end of the trial to explore participants’ experiences using semi-structured telephone interviews with a purposive sample of participants from both trial arms. Normalisation Process Theory informed data collection and analysis. Analysis involved directed content analysis within a Framework Approach.

**Results:**

Nineteen participants took part. They understood the purpose and requirements of the trial and identified beneficial outcomes from taking part. For the majority, the work of the trial was easily accommodated into daily routines and did not require new skills. There were mixed views about the value of the information provided by RESTORE, depending on time since diagnosis and treatment. Personal factors, constraints of the intervention, and environmental context inhibited the integration and embedding of RESTORE into everyday life. Access to the intervention at an early stage in the treatment trajectory was important to effective utilisation, as were individual preferences for delivery of information.

**Conclusion:**

The theoretical foundations of the intervention were sound. Participants derived benefits from the intervention but barriers to implementation and integration suggest that RESTORE and the trial processes require some modification before testing in a full trial.

**Trial registration:**

ISRCTN67521059 (10^th^ October 2012).

**Electronic supplementary material:**

The online version of this article (doi:10.1186/s12911-015-0214-y) contains supplementary material, which is available to authorized users.

## Background

Process evaluations are an important part of randomised controlled trials (RCT), particularly complex healthcare interventions [1–5]. They increase understanding of participants’ views of the intervention and trial processes, so that they can be modified prior to a large-scale trial. A theoretical approach to a process evaluation supports understanding and explanation of the processes involved during the implementation of an intervention and its potential integration into everyday practice [6].

This paper reports findings from a qualitative process evaluation of RESTORE, an exploratory RCT of a web-based intervention to enhance self-efficacy to manage cancer-related fatigue (CRF) following primary cancer treatment [7]. CRF is one the most common and distressing symptoms experienced by people affected by cancer with no known effective pharmacological treatment [8]. There is growing support for physical activity [9] and interventions that include psychosocial support and cognitive behaviour therapy in improving CRF [10], however access to existing programmes is limited and such programmes are, most often, resource intensive.

Access to the internet is growing rapidly, particularly amongst older adults [11] and increasingly the internet is being used to promote health behaviour change [12] and deliver self-management interventions for long-term conditions [13, 14]. Two existing publications describe web-based interventions to improve CRF [15, 16]. However none have considered the importance of self-efficacy to manage CRF or include any evidence of process evaluation.

Full details of the development, content and trial protocol of RESTORE have been published elsewhere [17]. Briefly, the content of RESTORE was theoretically informed [18, 19]. It consists of 5 sessions, completed independently by participants, at weekly intervals, bringing together clinical knowledge and lay examples from survivors. Sessions 1-2 are compulsory and introduce CRF (including prevalence, definitions and aetiology), and the concept of setting SMART goals. For the following 3 weeks participants then choose from sessions on i) diet, sleep, exercise, home and work life; ii) thoughts and feelings; iii) talking to others. Participants can complete all sessions, or spend more time on a session/s most relevant to them. A choice of structured activities, such as goal setting and keeping a fatigue diary are provided throughout the intervention, in addition to automated tailored feedback on achievement of goals and video clips of patients’ stories.

During the exploratory RCT participants, who had completed primary treatment within the last 5 years, were randomised to receive either RESTORE or a leaflet comparator.

The process evaluation was conducted at the end of the trial. The aim was to understand the work required for participants; establish if the concept and theoretical foundations of the intervention were sound; identify barriers to integrating and embedding the intervention into everyday routines; and ascertain whether and how implementation needed to be improved.

## Evaluation approach

Normalisation Process Theory (NPT) provided the theoretical lens for the evaluation [20, 21]. NPT is an action theory concerned with what people do rather than their views and beliefs. By focusing on collective, distributed patterns of action NPT seeks to explain how and why things become, or do not become, embedded into everyday practice [21]. NPT assists “in understanding and explaining the dynamic processes that are encountered during the implementation of complex interventions and technological or organisational innovations in healthcare” [5].

In NPT, 4 theoretical constructs shape the processes of successful intervention implementation. These dynamic and interrelated domains are: coherence – the sense-making work to understand the extent to which an intervention is meaningful, achievable and necessary; cognitive participation – the relational work that promotes or inhibits participants’ enrolment and legitimation of a complex intervention; collective action – the enactment work that bring the intervention into use; and reflexive monitoring – the appraisal work carried out to assess the effects of the intervention.

NPT informed the design and development of the evaluation and was applied to data collection and analysis. The decision to use NPT was deliberate as it offered a verifiable and empirically grounded model of the factors promoting or inhibiting the routine incorporation and embedding of the RESTORE intervention into participants’ lives and a theoretical framework for understanding and evaluating implementation as a process. NPT has been mainly used in qualitative studies of implementation [22], including systematic reviews [23–25]. Spangaro et al used NPT to evaluate the implementation of a complex intervention – a screening tool – to identify women at risk from intimate partner violence [26] and the use of NPT in a process evaluation of a trial of supported self-management for long term conditions in primary care helped to explain the outcomes of the study [27]. Qualitative research review (RATS) guidelines were followed [28].

## Methods

### Evaluation design

An in-depth qualitative approach using semi-structured telephone interviews explored feasibility and acceptability by capturing the work involved in being a participant, and issues around the intervention such as: implementation; usage; satisfaction and setting. Ethical approval was gained from the NRES Committee South Central – Oxford A, and research governance approval from the appropriate NHS Foundation Trust, REF: RHM CAN0875.

### Participants

At the time of sampling, 81 participants in the trial had agreed to be interviewed for the process evaluation. From those who had indicated a willingness to take part a maximum variation sample was drawn from both trial arms across the 12 NHS Trusts to ensure participants with a range of characteristics were included, e.g. gender, age, and adherence to the intervention. A letter and information sheet was sent to potential participants who were asked to contact the research team if they were still prepared to take part. All participants gave written informed consent prior to the interview taking place.

### Data collection

Interviews were conducted between August and October 2013 by MM who has experience of working with patients and other vulnerable groups and was not involved in delivery of the RCT. Interviews were recorded (with consent) and transcribed verbatim (pseudonyms replace names). Notes were made at the time of interview and written up immediately after. Interviews lasted 20 - 60 minutes. It is only possible to speculate as to factors leading to the variation in interview length, but this appears to have been influenced by: participant’s recall of their participation in the trial; strength of opinion on their participation and/or the intervention/comparator; level of fatigue. Interviews were conducted using a topic guide (see Additional file 1: Appendix 1 for intervention group and Additional file 1: Appendix 2 for comparator group) focusing on trial implementation, usage of RESTORE or leaflet, benefits of taking part in the trial, value of RESTORE and suggestions for improving implementation.

### Data analysis

Transcripts were analysed using directed content analysis within a Framework Approach [29]. Initially a systematic reading and re-reading of the transcripts, along with contextual notes, was undertaken to enable familiarisation and stronger understanding of the data set. An initial coding framework was developed from a priori issues, and using the 4 constructs of NPT, and emerging themes identified at the familiarisation stage. The latter were placed in an ‘other’ category to reduce the likelihood of making the data fit the theory. Interviews were coded by an experienced qualitative researcher (MM) by applying the thematic framework to the data using numerical codes. Discussions about the coding process took place between MM and CRM, who is responsible for the development of NPT. The coded data were then ‘charted’ into a framework using the headings from the thematic framework. This facilitated the next stage of the analytic process, mapping and interpreting the data to look for patterns, associations and meaning. This was undertaken by MM and reviewed and discussed with CRM. Use of NPT to inform development of the interview guide, data collection and interpretation of results also serves to minimise researcher bias. Quotes were selected on the basis that they provided evidence of the interpretation of the data being made and a deeper understanding of the strength of feeling participants’ expressed about particular issues.

## Results

### Sample characteristics

One hundred and sixty three participants took part in the RCT, 85 were allocated to RESTORE and 78 to the Macmillan Cancer Backup ‘Coping with Fatigue’ information leaflet. See Foster et al [30] (under review) for a full description of the results of the RCT.

From the eighty one participants who had agreed initially to participate in an interview at the time of sampling, twenty two responses were received. Three of these declined to be interviewed. Most were female, < 60 years of age, with over half in the 50-59 age range. A range of cancer types were represented and most had breast cancer. See Table 1 for participant characteristics.

**Table 1:** Participant characteristics

Characteristic	Number of participants (*n* = 19)
Age	
<60 years	14
≥ 60 years	5
Gender	
Male	4
Female	15
Cancer type	
Breast	12
Colorectal	2
Head & Neck	3
Liver	1
Prostate	1
Trial Arm	
RESTORE	8
Leaflet	11
Adherence to intervention (*n* = 8)	
≤2 sessions	2
≥3 sessions	6

### Making sense of participation in the trial

Previous studies have shown that participants’ understanding of their involvement can affect trial outcomes and influence their views on its validity [31].

Most participants were able to identify beneficial outcomes from taking part. A number made changes to their behaviour and lifestyle as a result of using RESTORE or the leaflet which had led to a positive and personally relevant outcome:

*It [RESTORE] was the explanation of the different factors which effect fatigue that was positive as well because that changed behaviour, eating less chocolate (laughs) you know so, it had a positive effect in changing behaviour as well.* (Alison)

For others, involvement offered an opportunity to reflect on their fatigue, and re-evaluate what they could do. Several participants described learning to accept and realise their limitations:

*It [RESTORE] does make you think so I suppose taking things into consideration a little more, you know, and not being quite so hard on yourself. I think that was one of the big things because it, you know what I mean, I did tend to beat myself up and oh yes, you’re not like you used to be.* (Helen)

Some participants felt they had gained a better understanding of fatigue and how to manage its symptoms:

*But again it comes back to the RESTORE online piece that made me think about other avenues and how I can get more information, who I can go to, is this right … and all those sorts of things, so it helped me personally in a far wider range of activities and understanding of the condition I’ve got than I possibly would have done if I hadn’t done the RESTORE programme.* (Michael)

For some participants, benefits were derived specifically from the structured activities within RESTORE. Seven participants identified positive outcomes. The fatigue diary helped them gain a more informed understanding of their CRF, recognise patterns and triggers for their fatigue and provoked self-reflection of personal behaviour. Similarly, goal setting prompted self-reflection about lifestyle and behaviour and was considered one of the most useful elements of RESTORE.

*The goal setting was, to me, one of the best because it did just confirm to me and just make me re-think again, you know, that it was foolish to push myself, because I would absolutely push myself and then be wrecked for a couple of days whereas you know if you kind of pace yourself it was just, it was easier.* (Helen)

A number of participants derived benefits from their participation in the trial rather than RESTORE or leaflet per se. For example, several described feeling supported and reassured that there was interest in their condition and ‘*somebody is fighting your corner’* (Angela). For others, participation provided a focus and source of motivation previously absent:

*It’s actually given me something to do. I’m struggling, again I’m struggling, concentrating on things so that helped, because you know it’s given me something that I’ve got, you know I’ve got to finish, so that’s helped me.* (Stuart)

Completing the questionnaires also provided benefits, such as an opportunity to reflect on progress, or reassurance that fatigue was a ‘normal’ symptom. For those who had not experienced beneficial outcomes from RESTORE, the questionnaire was described as having the most impact. For these participants, it is possible that participation in the trial was the intervention, rather than RESTORE.

As a result of trial participation, most respondents reported increased confidence to self-manage their fatigue and considered their fatigue to be less bothersome.

### The nature of participation: the work of the trial

NPT aims to understand the work required for an intervention to be implemented and identify factors that promote and inhibit implementation.

#### Incorporating the trial into everyday routine

Most intervention group participants had accessed the 5 RESTORE sessions (compared to 43 % of the intervention group in the total RCT sample), and all had completed the first 2 mandatory sessions. Five/11 participants in the comparator group had accessed RESTORE upon completion of the trial. The overall length of the study was acceptable to most who found the 12 week duration ‘about right’. Most agreed that they had sufficient time to work through the sessions and complete questionnaires.

Most participants from both groups felt that the trial fitted into their daily lives. For many, adjustments were only needed due to behaviour or lifestyle changes made as a result of RESTORE or the leaflet. Participants were able to undertake the required aspects of the study at a time that was convenient for them without it being overly intrusive or disruptive. However, some had difficulty incorporating specific aspects, such as keeping a fatigue diary, into their routine.

#### Using the intervention

None of the participants reported having to learn new skills to use RESTORE. The general consensus was that it was straightforward and easy to operate, instructions were clear and simple to understand, and had been designed to be accessible, including those with lower levels of literacy and knowledge of the internet.

Few participants had encountered problems navigating the RESTORE website. However, some had experienced issues with functionality, for example, there were difficulties logging on; passwords being refused, screens freezing or closing down half way through. There were some instances of participants having to re-start sessions or fill in questionnaires more than once because they had failed to save; others reported receiving erroneous alerts about non-completion of sessions or questionnaires. While these difficulties did not appear to detract from users’ experience of RESTORE, it created additional work for several participants. Completing a duplicate questionnaire also caused some anxiety regarding the need for consistent responses.

#### Content of the RESTORE sessions

Most participants considered the language and terminology of the RESTORE sessions simple and accessible, did not assume any prior clinical knowledge, and were inclusive of those with lower levels of education.

There were mixed views about the information content of the sessions. Some learned useful hints and tips to deal with the symptoms of CRF, and liked the succinct information. Others felt the information was too simplistic, too generic, did not offer anything new and would have preferred signposting to resources offering more specific information.

The comparator group expressed similar views regarding the leaflet.

#### Completing outcome measures

Few participants found the questionnaires at 3 time points burdensome. Several participants who were ≥18 months post diagnosis felt some questions were not relevant. For example, items about health service use and seeking help from health professionals were more suited to those with a current diagnosis and were an unwelcome reminder of potential problems they may encounter. It was suggested that questions should be specific to cancer types and time since diagnosis.

Several participants considered the psychological aspect of cancer was missing and should be included in the questionnaires. They would have welcomed an opportunity to share how their cancer, and the associated fatigue, impacted on them emotionally.

Several participants were aware the questionnaires requested the same information more than once. For some this was a source of anxiety and revealed additional decision-making work spending time deliberating over responses, worried that inconsistent answers could affect the results of the trial.

### Barriers to integrating and embedding RESTORE into everyday life

A central aim of the process evaluation was to identify barriers to completing the intervention. Effective implementation refers to how workable it is in the everyday routines of participants. Applying NPT, factors were identified that inhibited the integration and embedding of RESTORE into everyday life. These are presented in Table 2 and relate to the agent (user), the context, and the object (intervention).

**Table 2:** Barriers to integrating and embedding the intervention

Factor	Examples	Empirical evidence
The agent:		
Intervention does not seem relevant	Participant did not consider their fatigue level sufficiently high to require intervention.	*It wouldn’t have been that helpful because I wasn’t … it (fatigue) wasn’t that often. It wasn’t as though every day I had, it was a struggle, not like for some people … I wasn’t affected as bad as some people. (Robert)*
	The intervention did not offer anything novel or innovative. Information was not deemed useful.	*I just thought it was an extension of the booklet, I didn’t see it in any other way. (Georgina)*
	Participant engaged as a research volunteer in a trial and was not looking for strategies to manage fatigue symptoms or significant personal benefit from involvement in the study.	*I wouldn’t say that I was expecting to gain a lot of insights into how to deal with the fatigue, more a sense of somebody out there is needing people for this [study] and I’ll be helpful. (Emma)*
Intervention requires skills that user does not have (or are limited)	Participant dislikes/distrusts IT/online nature of intervention/lacks confidence to use IT/internet.	*I’ve just got basic skills because I’ve never really, I’m not somebody that really sits in front of the computer. I’ve never been one of them. (Iris)*
The context:		
Intervention is not easy to fit in to daily life	Using the intervention was too much to do at a time when participant is fatigued.	*Sometimes I couldn’t be bothered … it was just making the effort to go on and do it. (Angela)*
	Accessing intervention requires additional ‘work’ or making adjustments to routine.	*My home computer had broken … so I had to do it using the work computer and I’d only just gone back to work and I wasn’t working very many hours so I had to tag it on either, come in early and do it at the beginning of my working day or tag into the end of my working day. (Laura)*
	Participants found it difficult to accommodate or ‘fit in’ using the intervention on a day-to-day basis.	*Int: One of the suggestions was to keep a fatigue diary, is this something that you did?*
		*Res: No I didn’t on the basis I was too busy (Sylvia)*
The object:		
Intervention has unintended negative impact	Using the intervention is a reminder of cancer/being a cancer survivor.	*And I think I might also feel, I’m concentrating, the more I concentrate on this, the more I would almost be looking for symptoms? And I suppose it’s also that sense of wanting to kind of move on from it as much as possible … it would be a daily reminder and I think that might be at because of where I am … it was five years ago that I had the diagnosis and I had the surgery. (Emma)*
	Interaction with the intervention makes participant question if they are really fatigued.	*I tell you what was a little bit, made me feel a little bit funny … was that knowing that some people were a lot worse than me, I thought have I just been wimping here (laughs). (Angela)*

### Reflecting on RESTORE and trial participation: appraisal work

As part of the process of embedding an intervention into their everyday lives, participants engage in reflexive monitoring [21]. This involves making judgements about its usefulness, assessing its effectiveness, as well as how the intervention and the trial processes might be improved to facilitate future implementation.

#### Timing of participation in relation to completion of treatment

Half of participants felt that the timing of participation was ‘about right’, with the other half indicating they would have preferred it sooner. Those who would have preferred to have taken part sooner were at least 1 year post diagnosis (most ≥3 years post diagnosis and treatment). Reasons cited included: lack of awareness and information about CRF; needing advice and suggestions for managing CRF; and reassurance that how they felt was ‘normal’:

*It would have been nicer earlier … because I think it was for the first couple of years that I really kind of struggled the most … it really was the first couple of years that I really had a really hard time … I hadn’t received any information at all, I mean the information that I received was all the kind of you know, the lumpectomy and the chemo and the radiotherapy, all that side of… and then it sort of, the information stopped … I got nothing. (Helen)*

#### Comparing the intervention with the comparator: RESTORE vs Leaflet

At the end of the trial, participants in the comparator arm were given access to RESTORE. Reasons for not accessing RESTORE centred on 3 factors: dislike of computers; believing RESTORE was an extension of the leaflet that did not offer anything new; lack of awareness of RESTORE’s availability.

Of those who had accessed the leaflet and RESTORE, several preferred RESTORE finding it more flexible and convenient to use, particularly the online nature and access via a mobile phone. In addition, it offered increased opportunities to ‘dip in and out’, which encouraged participants to work their way through the sessions when it suited them. This was in contrast to the leaflet which they felt compelled to read in one sitting:

*I liked the way it broke it up so that you would do a bit one week and a bit later on, rather than with the booklet that I read it all at once, you feel as though you’ve got to do it all at once, whereas [RESTORE] was more timely, perhaps that’s what made it easy to get into*. (Sally)

Participants liked the interactivity of RESTORE which brought the information alive through video links to patient stories, and was generally perceived to be more engaging than the leaflet. This motivated some participants to take on board, for the first time, the advice and suggestions offered, or try the structured activities. As one participant explained:

*… it hadn’t dawned on me to actually try, to try them, so actually doing so and setting the targets and having to put them down as an, even if there is no other person involved, the fact of actually putting them online and you’re going to be shown them again in a week’s time is more engaging than just scribbling it on a piece of paper and then not remembering where you’ve put it.* (Amanda)

Some felt the leaflet was more convenient and easier to access because it was immediately available, could be consulted anywhere, and at any time. It was a welcome change to using a computer. It was also evident that for some participants the leaflet required less work.

#### Improving the intervention

Few participants identified ways in which RESTORE could be improved. One participant suggested including more pictures and graphics to enhance the content of the RESTORE pages. This could include cartoons. Recent research has shown the value of humour, and particularly the use of cartoons, in helping people with long term conditions adjust and cope with their illness, and of their potential to improve the narrative and tailoring of information to support self-management [32].

A second suggestion was ensuring equality of access to the intervention. One participant was concerned that it excluded certain groups on both a financial and demographic level. For example, using RESTORE necessitated owning a computer, or at least being able to access the internet, which was felt to exclude some socio-demographic groups.

Similar concerns were expressed regarding equality of access for those with learning difficulties or cognitive disorder. While it was acknowledged that in its current format RESTORE was simple and straightforward to use, it would still pose challenges to those with even mild cognitive impairment.

## Discussion

The process evaluation has increased understanding about the feasibility and acceptability of RESTORE and the trial processes and will inform the design of a future definitive trial. Using NPT to develop and shape the analytical framework enabled identification and provided insights into the different kinds of work that participants engaged in to bring the intervention into everyday use, as well as the factors that impeded implementation and integration.

A number of issues were important to participants and influenced the extent RESTORE was assimilated into their daily lives. The majority of participants were able to identify beneficial outcomes, including increased confidence to manage the effects of CRF. However, for some participation in the trial was the intervention.

Most of the work required to use RESTORE and take part in the trial was incorporated relatively seamlessly into participants’ lives and could be attributed to: the acceptability of the study duration; sufficient time being given to complete the sessions; and convenience of the intervention which enabled users to access it at a time that suited them. Usability of RESTORE was important to implementation as participants were able to draw on existing skills without the need to develop new ones. This could be influenced by the socio-demographic characteristics of the sample, most of whom were confident internet users. Recent research indicates that the problem of internet access is diminishing, even amongst older people [33]. Further consideration will need to be given to the extent to which those with limited IT skills would be able to use RESTORE with the same ease without help and support.

Mixed views about the information content of RESTORE suggest the types of material included may need to be re-visited for the future design of the intervention. The need for less generic and more specific information was considered important. While RESTORE needs to retain a broad reach, improved signposting to resources dealing with a variety of cancers and relevant to users at various distances from diagnosis and treatment, and inclusion of more wide-ranging patients stories, offer some ways RESTORE could be tailored to address the informational needs of a diverse range of users. This could reduce the potential for information to be viewed as an unwelcome reminder of their cancer.

Effects of interventions may be determined by individual preferences of format, i.e. some prefer the web-based resource and others a hard copy, therefore there may be merit in identifying early on which format best suits users and employing an alternative means of testing the effectiveness of RESTORE, through for example a patient preference trial [34, 35].

Views derived from the appraisal work carried out by participants revealed how RESTORE and trial processes could be improved to facilitate future implementation. One of the most significant findings was timing of participation in relation to completion of treatment and providing access to the intervention at an early stage. A recent systematic review, to develop practice guidelines to inform healthcare providers about screening, assessment and effective management of CRF in adults, reported all patients experiencing CRF should receive preparatory education early in the disease and treatment trajectory which should form part of routine care for patients affected by CRF [36].

The ability to access information and advice on managing CRF, engaging in goal setting and monitoring of CRF during the early stages was felt to be essential to the effectiveness of the RESTORE. This highlights the importance of identifying a ‘teachable moment’ [37–39], that is the opportune time to deliver the intervention. This may vary, but is likely to be in the early stages of the recovery trajectory when cancer survivors are looking to return to their previous lifestyle and making positive changes to their quality of life. Recruiting participants to a future definitive trial within one year of treatment completion may achieve the most impact for participants. This would enable it to fit with the timing of current survivorship care packages, highlighting strategies to deal with fatigue if it persists past initial recovery from treatment.

### Study limitations

It is important to acknowledge the potential limitations to this study. Only a small number of participants from the intervention group were included. It is only possible to speculate why more participants were not willing to be interviewed. Those who came forward had largely positive views of the intervention and most in the intervention group had accessed all 5 sessions compared with 43 % of the total RCT sample. It is possible those who declined had not received positive benefits but were unwilling to share these, particularly if they believed their experience to be unrepresentative. However, this was a study whose participants were fatigued and for some an interview might have been an additional task they felt unable to undertake.

## Conclusion

Findings from the process evaluation confirm that the theoretical foundations of RESTORE are sound. Several barriers and facilitators to implementation and integration have been identified. There are a number of factors that are likely to influence ‘normalisation’ and moderate the constraints of the intervention. Central to this is the self-perception of the user, their adjustment to their identity as a cancer survivor, and the temporal distance from diagnosis, which either strengthens or undermines the relative advantage of the intervention. This suggests that RESTORE may be more effective for those in the early stages of the treatment trajectory or more at ease with their cancer survivor identity.

## Additional file

Additional file 1:
**Interview guides.** (DOCX 21 kb)

## Abbreviations

NPT: Normalisation Process Theory
RCT: Randomised controlled trial
CRF: Cancer related fatigue
RATS: Qualitative research review guidelines
