# Supplementary material for: RESTORE: an exploratory trial of a web-based intervention to enhance self-management of cancer-related fatigue: findings from a qualitative process evaluation
Source: BMC Med Inform Decis Mak. 2015 Nov 14;15:94. doi: 10.1186/s12911-015-0214-y (PMC4650501; doi:10.1186/s12911-015-0214-y)
Supplement: Additional file 1: — Interview guides. (DOCX 21 kb) [file 12911_2015_214_MOESM1_ESM.docx]

Appendix 1: Participant Telephone Interview Guide: Intervention Group

1. **Introduction**

Interviewee consent – discuss interviewee’s consent to be interviewed:

- Check interviewee is happy to go ahead with the interview
- Go through consent form (emphasise confidentiality)
- Check interviewee is happy for interview to be audio-recorded
- Ask if they have any questions or concerns before proceeding

Purpose of the interview – We are interested in hearing your opinions on a number a things and having your views both on taking part in a study, and using the online RESTORE resource. For example, we would like to ask you about what is has been like to be involved in the study, how you feel you were involved in the research process, your thoughts on using the RESTORE resource and any difficulties you faced; if you think it has helped you and any recommendations for improvements.

**2. Involvement in the study**

i. Can you begin by telling me how you came to be involved in the study?

ii. What was your motivation for taking part? Why did you decide to become involved?

iii. Did you have any specific aims in taking part? Was there anything you hoped to get out of being involved?

- Did your involvement in the study meet any expectations you might have had?

iv. Can you describe what taking part in the trial has involved?

- - Have you had to do anything different or make any adjustments in your day to day life as a result of being part of the study? If so, in what ways? If no, could you explain why?
  - One of the suggestions of the study was to keep a fatigue diary. Was this something that you did? If yes, how did you find doing this (e.g. time consuming, boring/waste of time, hard to remember to complete regularly)?
  - You were also asked to set regular goals. How did you find this?
  - How did you find having to complete questionnaires at different times during the study?
    - If you can remember, what are your views on the questions asked?
    - What did you think on the length of the questionnaire?

**3. The intervention: RESTORE/LIFEGUIDE**

i. We would like to know about your experience of using the online RESTORE resource. Could you tell me what your overall experience was?

- - What did you like most about it?
- What did you like least?
- Out of the five sessions which did you find the most useful and why?
- What would you say were the least useful sessions?
- Did you access all sessions?

For those patients who accessed few or none – can you tell me why this was? (e.g. technical problems, lack of time, too fatigued, info not helpful)

- Were there some sessions you accessed repeatedly?

ii. How did you find using RESTORE?

- Did you have any problems finding your way around the website?
- Was it clear what you had to do/were the instructions easy to understand?
  - Were there any terms or words used that you didn’t understand or were unclear as to the meaning?
  - What did you think about the pictures/graphics that were used?

iii. Do you think you need to have certain skills to be able to use RESTORE?

- If yes, what are these?
- Did you have to learn any new skills to be able to use RESTORE? (e.g. IT skills, use internet?
  - Where did you access RESTORE? (e.g. from own PC/tablet/phone; family/friend’s; library; internet cafe)

iv. Could you identify any barriers to using RESTORE? (e.g. logging in, accessing your account, finding somewhere private to use)

**4. Personal experience**

i. How did you find the overall length of the study?

ii. We are interested in gaining your views on the timing of being invited to take part in the study. How did you find starting it in relation to the completion of your treatment? (e.g. was it too soon, would you have preferred to have started sooner?)

iii. Do you feel you have benefited from taking part in the study? (prompts: feel more confident to manage fatigue; find fatigue less bothersome as result of using RESTORE; continued to use skills/activities, e.g. fatigue diary, goal setting)

iv. Do you have any suggestions as to how the study could be improved? (N.B. prompt for recommendations for RESTORE and research process).

v. Have you received feedback during the study? Do you expect to be informed on the results when the study has ended? How important is this to you?

vi. Do you feel you have an informed understanding of the purpose of the study?

**5. Anything to add**

- Is there anything you would like to add, or anything you would like to mention that you feel hasn’t been covered?

Appendix 2: Participant Telephone Interview Guide: Comparator Group

1. **Introduction**

Interviewee consent – discuss interviewee’s consent to be interviewed:

- Check interviewee is happy to go ahead with the interview
- Go through consent form (emphasise confidentiality)
- Check interviewee is happy for interview to be audio-recorded
- Ask if they have any questions or concerns before proceeding

Purpose of the interview – We are interested to hear your opinions on a number a things and having your views, both on taking part in a study, and using the *Coping with Fatigue* leaflet and the online RESTORE resource. For example, we would like to ask you about what it has been like to be involved in the study, how you feel you were involved in the research process, your thoughts on using the leaflet and the RESTORE resource once it became available and any difficulties you faced; if you think either has helped you and any recommendations for improvements.

**2. Involvement in the study**

i. Can you begin by telling me how you came to be involved in the study?

ii. What was your motivation for taking part? Why did you decide to become involved?

iii. Did you have any specific aims in taking part? Was there anything you hoped to get out of being involved?

- Did your involvement in the study meet any expectations you might have had?

iv. Can you describe what taking part in the trial has involved?

- - Have you had to do anything different or make any adjustments in your day to day life as a result of being part of the study?
  - One of the suggestions in the leaflet was to keep a fatigue diary. Was this something that you did? If yes, how did you find doing this (e.g. time consuming, boring/waste of time, hard to remember to complete regularly)?

v. For future work we would like to know your views on the questionnaires you were asked to complete at different times during the study. Could you tell me what you thought about:

- the length of the questionnaire

types of questions asked, did they ask for the same information more than once?

**3. The intervention: Coping with Fatigue leaflet/Accessing RESTORE**

i. We would like to know about your experience of using the leaflet. Could you tell me what your overall experience was? (e.g. useful, helpful, informative)

- - What did you like most about it?
- What did you like least?
- Which sections, if any, did you find the most useful and why?
- Which sections, if any, would you say were the least useful?
- Did you read all sections? Were there some you read more than once?

ii. On completion of the study you were able to access the online RESTORE intervention if you so wished. Did you do this?

- If yes, what are your views on RESTORE?
  - What did you like most about it?
  - What did you like least?
  - Were there sessions you thought were more useful than others?
  - Did you have any problems finding your way around the website?
    - Was it clear what you had to do/were the instructions easy to understand?
    - Were there any terms or words used that you didn’t understand or were unclear as to the meaning?
  - What did you think about the pictures/graphics that were used?
  - If no, why did you not access RESTORE? (prompt: limited access to internet; no interest in doing so; leaflet provided sufficient info; limited IT skills) **Go to Section 4**

iii. How did the RESTORE resource compare with the leaflet? (prompt: provided more info; liked interactive nature; too time consuming; need internet access; need more privacy)

iv. You were also asked to set regular goals. How did you find this?

**4. Personal experience**

**i.** How did you find the overall length of the study?

ii. We are interested in gaining your views on the timing of being invited to take part in the study. How did you find this in relation to the completion of your treatment? (e.g. was it too soon, would have preferred to have had it sooner)

iii. Do you feel you have benefited from taking part in the study? (prompts: have you incorporated any of what you read in the Leaflet/from RESTORE into your daily life, feel more confident to manage fatigue; find fatigue less bothersome as a result of reading the leaflet)

iv. Do you have any suggestions as to how the study could be improved? (N.B. prompt for recommendations for RESTORE and research process).

v. Have you received feedback during the study? Do you expect to be informed on the results when the study has ended? How important is this to you?

vi. Do you feel you have an informed understanding of the purpose of the study?

**5. Anything to add**

- Is there anything you would like to add, or anything you would like to mention that you feel hasn’t been covered?
